# Bacterial micro-aggregates as inoculum in animal models of implant-associated infections

**DOI:** 10.1016/j.bioflm.2024.100200

**Published:** 2024-05-09

**Authors:** Katrine Top Hartmann, Regitze Lund Nielsen, Freja Cecilie Mikkelsen, Bent Aalbæk, Mads Lichtenberg, Tim Holm Jakobsen, Thomas Bjarnsholt, Lasse Kvich, Hanne Ingmer, Anders Odgaard, Henrik Elvang Jensen, Louise Kruse Jensen

**Affiliations:** a Department of Veterinary- and Animal Sciences, University of Copenhagen, Grønnegårdsvej 7, 1870, Frederiksberg C, Denmark; b Costerton Biofilm Center, Department of Immunology and Microbiology, University of Copenhagen, Blegdamsvej 3B, 2200, Copenhagen, Denmark; c Department of Clinical Microbiology, Copenhagen University Hospital, Rigshospitalet, Blegdamsvej 9, 2100, Copenhagen, Denmark; d Department of Orthopedic Surgery, Copenhagen University Hospital, Rigshospitalet, Inge Lehmanns vej 6, 2100, Copenhagen, Denmark

**Keywords:** Biofilm, Bacterial aggregates, Infection, Implant-related infections, Animal model

## Abstract

Is it time to rethink the inoculum of animal models of implant-associated infections (IAI)? Traditionally, animal models of IAI are based on inoculation with metabolically active overnight cultures of planktonic bacteria or pre-grown surface-attached biofilms. However, such inoculums do not mimic the clinical initiation of IAI. Therefore, the present study aimed to develop a clinically relevant inoculum of low metabolic micro-aggregated bacteria. The porcine *Staphylococcus aureus* strain S54F9 was cultured in Tryptone Soya Broth (TSB) for seven days to facilitate the formation of low metabolic micro-aggregates. Subsequently, the aggregated culture underwent filtration using cell strainers of different pore sizes to separate micro-aggregates. Light microscopy was used to evaluate the aggregate formation and size in the different fractions, while isothermal microcalorimetry was used to disclose a low metabolic activity. The micro-aggregate fraction obtained with filter size 5–15 μm (actual measured mean size 32 μm) was used as inoculum in a porcine implant-associated osteomyelitis (IAO) model and compared to a standard overnight planktonic inoculum and a sham inoculum of 0.9 % saline. The micro-aggregate and planktonic inoculums caused IAO with the re-isolation of *S. aureus* from soft tissues, bones, and implants. However, compared to their planktonic counterpart, neither of the micro-aggregate inoculated animals showed signs of osteomyelitis, i.e., sequester, osteolysis, and pus at gross inspection. Furthermore, inoculation with low metabolic micro-aggregates resulted in a strong healing response with pronounced osteoid formation, comparable to sham animals. In conclusion, the formation and separation of low metabolic bacterial micro-aggregates into various size fractions is possible, however, planktonic bacteria were still seen in all size fractions. Inoculation with micro-aggregates caused a less-aggressive osteomyelitis i.e. combination of infected tissue and strong healing response. Therefore, the use of low metabolic micro-aggregates could be a relevant inoculum for animal models of less-aggressive and thereby slower developing IAI and add in to our understanding of the host-implant-bacteria interactions in slow-onset low-grade infections.

## 1 Introduction

The use of medical implants such as prosthetic joints, hernia meshes, orthopaedic plates and screws, breast implants, artificial heart valves, and urinary and vascular catheters is increasing [1]. Implantable devices have certain infection rates depending on the specific device [1] and, thus, because of the growing number of implanted materials used in modern medicine, an increase in bacterial implant-associated infections (IAI) is seen [2]. Even though a shift away from animal models has lately been observed, they are still indispensable for studies of pathogen-host-implant interactions in IAI [3] and for testing new treatment strategies intended for human use [3]. The goal of an animal model is to replicate the human disease as closely as possible, and several models of IAI have been developed based on bacterial inoculation of different animal species.

In animal models of IAI the inoculum *per se* is equally important as both the animal and the implant. The inoculum constitutes the bacterial strain, dose, and volume into which the bacteria are suspended [4]. In addition, the metabolic state [5] and the organization of the bacteria [6] are equally important factors to consider.

**Fig. 1:**
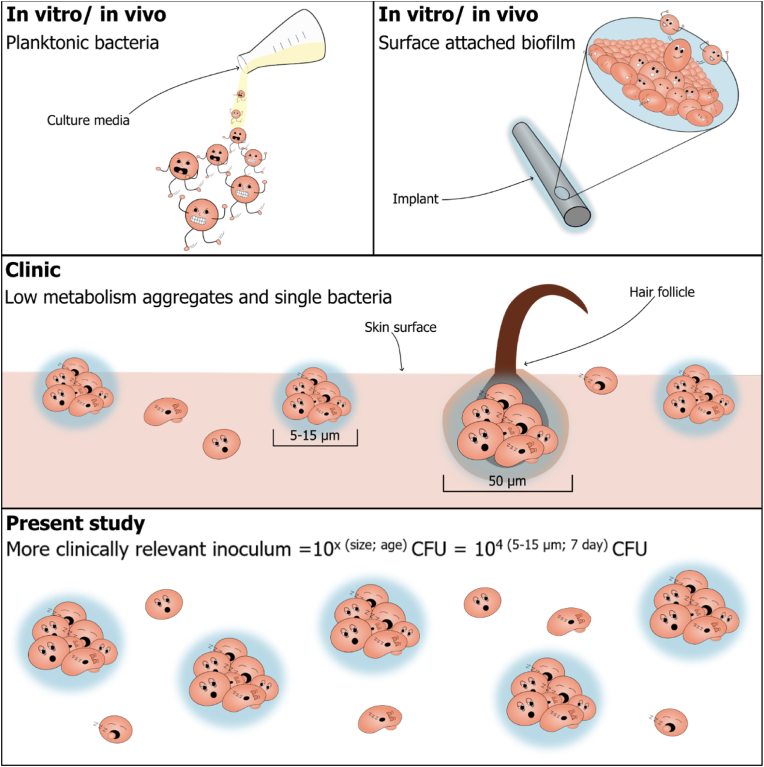
Illustration of the different forms of bacterial inoculums commonly used in animal models, compared to the bacteria causing clinical implant-associated infections (IAI). Top left: Freshly grown planktonic bacteria from overnight cultures with good nutrition and environmental conditions supporting their growth. Top right: Surface attached biofilm, pre-grown on the implant prior to insertion in the animal model. Middle: Bacteria on the skin surface causing IAI by contagious spread during implantation in clinical infections. The bacteria are both found as aggregated bacteria in varying sizes depending on their localization, i.e., on the skin surface or within the hair follicles and as single scattered bacterial cells, all with a decreased metabolic activity. Bottom: Aggregated and single scattered planktonic bacteria of low-metabolic activity separated into size fractions from seven-day-old cultures and used as inoculum in the present study. As an additional finding this paper proposes a new definition for bacterial concentrations, including both CFU count as well as size and age/culturing time in the following way: Concentration ^(size; age)∼^10^4 (5–15 μm; 7 day)^ CFU.

Most often, overnight cultures of planktonic bacteria are used as inoculum in animal models of IAI [7]. Inoculation with planktonic bacteria may be representative of acute infections [6], but IAI are typically chronic biofilm-based infections developing slowly. Thus, IAI are not caused by high doses of metabolically active planktonic bacteria. As an alternative, several animal models of IAI have been based on inserting implants or hydrogel beads with pre-grown mature biofilm [6]. However, an inoculum based on implants with pre-grown biofilm does not equalize the pathogenesis of IAI (Fig. 1).

IAI is caused by bacteria entering deeper tissues during implantation or by a later hematogenous spread [8]. Contagious infection is reported as the most common, and the bacteria typically originate from the skin surface [8]. On the skin surface, bacteria are present as relatively inactive single bacteria or small non-attached biofilm aggregates [9] and thus resemble neither highly active planktonic bacteria nor pre-grown surface-attached biofilms [10]. Biofilms are recalcitrant toward the immune system and antimicrobials [11] due to a combination of factors such as the protective matrix covering the aggregates [12,13], the size of the aggregates [14,15], the metabolically inactive state of the bacteria [16,17], and their differentiation into distinct phenotypes inside the aggregates [14,18,19]. Studies have shown that skin surface aggregates can persist even after surgical preparation and disinfection of the skin [20,21] and that the average diameter of aggregates on the skin surface is 5–15 μm in diameter, whereas aggregates located in hair follicles are larger with an average size of 50 μm in diameter [9].

The present study aimed to develop a protocol for establishing an inoculum for the induction of IAI in animal models based on low metabolic bacterial micro-aggregates resembling those found on the skin of humans. Controlled, seven-day-old bacterial micro-aggregates of a well-described porcine *Staphylococcus aureus* strain [22] were produced and separated into fractions of different sizes. The sizes of the micro-aggregate fractions were evaluated by microscopy, and their metabolic activity was assessed using isothermal microcalorimetry. Finally, micro-aggregate fraction obtained with filter size 5–15 μm were used for inoculation in a porcine model of implant-associated osteomyelitis (IAO), and the pathology was compared to pigs infected with a standard inoculum of planktonic bacteria. The micro-aggregates caused a less aggressive IAO, with a pronounced bone healing response compared to their planktonic counterpart. This indicates that low metabolic micro-aggregates cause slower-developing low-grade infections, more resembling clinical cases of chronic IAO in comparison to inoculation with planktonic bacteria.

## 2 Methods

### 2.1 Culturing

*S. aureus* strain S54F9 (*spa*-type t1333), originally isolated from a porcine lung abscess was used in the present study. This strain was chosen since it is known to cause infections in porcine models [[23], [24], [25]], including osteomyelitis in a highly used IAO porcine model [[26], [27], [28]]. The strain has previously been whole-genome sequenced [22] and is capable of biofilm formation [29]. Tryptone Soya Broth (TSB) has previously been reported to support *S. aureus* aggregation [30]. Biofilm micro-aggregates were produced by culturing S54F9 in 400 ml TSB (Oxoid CM0129B, Oxoid Ltd, Basingstoke, United Kingdom) in a shaking incubator at 37 °C and 150–180 rpm. for seven days. Planktonic overnight cultures of S54F9 were grown in Lysogeny Broth (LB) (Difco no. 240230, Becton, Dickinson and Company, New Jersey, USA) and TSB in a shaking incubator at 37 °C and 150–180 rpm. for 24 h. The planktonic bacteria cultured in LB has previously been used as planktonic inoculum in the IAO porcine model [[26], [27], [28]] and was therefore used as planktonic inoculum in the present study. The planktonic bacteria cultured in TSB were included for comparison of differences between the different growth media and culturing times.

### 2.2 Separation of micro-aggregates into different size fractions

**Fig. 2:**
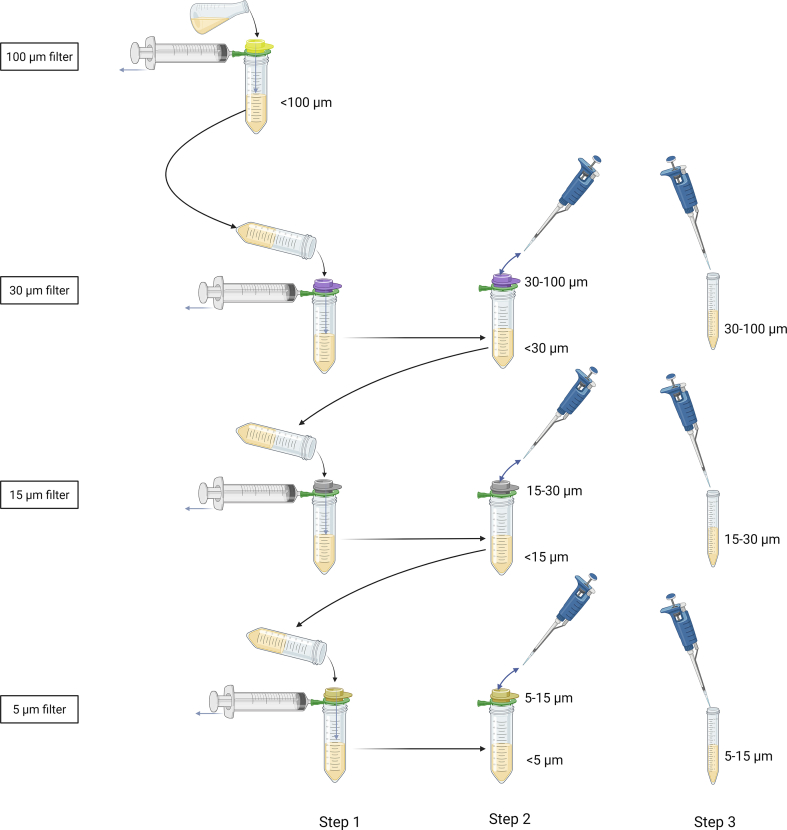
Overview of the filtration process of the bacterial micro-aggregates. The aggregated seven-day-old Tryptone Soya Broth (TSB) culture was filtered through cell strainers of different pore sizes to separate the aggregated bacteria according to size. Starting with the filter of the largest pore size moving downwards. After filtration (Step 1), the micro-aggregates captured at the top of the filter were resuspended in sterile isotonic saline (Step 2) and recollected to a separate tube (Step 3). Created with BioRender.com.

Bacterial micro-aggregates were separated into fractions of different sizes by cell strainers (pluriStrainer, Pluriselect, Leipzig, Germany), as described in a recent study with modifications [31]. The seven-day-old TSB culture was filtered through four different cell strainers with a pore size of 100 μm, 30 μm, 15 μm, and 5 μm, respectively, i.e., starting with the filter of the largest pore size moving downwards (Fig. 2). Vacuum was applied using a 50 ml syringe connected with luer lock to a connector ring, between a 50 ml falcon tube and the cell strainer. Following filtration, micro-aggregates were collected on the top of the filters by adding 5 × 1 ml of isotonic sterile saline, washing the filter and suspending the micro-aggregates into saline. The dispensed bacterial micro-aggregates were recollected into sterile 10 ml centrifuge tubes. Three different fractions of micro-aggregates were collected from the top of the filters and named accordingly to the filter sizes used for obtainment: 5–15 μm, 15–30 μm, and 30–100 μm. The fraction that was filtered through the 5 μm filter and planktonic overnight cultures were collected and diluted 1:9 with isotonic sterile saline to prevent further growth until colony-forming units per milliliter (CFU/ml) were estimated and dilution to the final inoculum concentration was made.

### 2.3 Estimation of CFU/ml

Enumeration of CFU/ml was estimated for all four fractions of micro-aggregates (<5 μm, 5–15 μm, 15–30 μm, 30–100 μm) as well as for the planktonic overnight cultures in TSB and LB, by serial dilution and plating of 50 μl of dilutions on blood agar plates. The blood agar plates were incubated for 24 h at 37 °C under normoxic conditions, followed by the CFU/ml determination by plate count method [32]. Ultrasound sonication was applied to break up micro-aggregates to better estimate CFU/ml of the 5–15 μm micro-aggregates [30]. Aliquots of 1 ml 5–15 μm micro-aggregates were subjected to probe sonication. The sonicator (Bandelin sonopulse HD2070/UW2070, Bandelin Electronics, Berlin, Germany) was fitted with a MS 73 probe (highest amplitude at 100 % = 212 μm_ss_) and the samples were subjected to pulses of 500 ms. The amplitude (power) and number of cycles (pulses) were varied, in the range of 50–90 % in power and 15–25 pulses, to map the effect of sonication intensity. Subsequent to sonication, the samples were plated in 10 μl spots (three technical replicates) on Tryptic Soya Agar (TSA) plates, and colonies were counted the following day. Treatment of 5–15 μm micro-aggregate fraction with different sonication settings showed no differences in CFU/ml compared to non-sonicated controls. Therefore, sonication was not applied any further when estimating the CFU/ml.

### 2.4 Visualization of micro-aggregates by light microscopy

All fractions of micro-aggregates and planktonic overnight cultures were visualized by light microscopy. A 1 ml aliquot from each sample was centrifuged at 4500 rpm for 10 min. The supernatant was removed, and the pellet was turned a few times. Two 50 μl droplets of the pellet were placed on adhesive objective glasses using sterile pipettes and left for flat drying for 1 h. After drying, the samples were spray-fixed with 96 % ethanol and left for flat drying for 20 min. The samples were stained with a combined staining technique based on immune histochemical (IHC) staining directed towards *S. aureus* and histochemical staining with Alcian Blue coloring carbohydrates blue [29]. In each droplet, areas with only a single cell layer were identified, the five largest micro-aggregates were selected, and pictures were taken on a light microscope (Olympus BX60) (objective 40x/0.75). The micro-aggregate size was subsequently measured using ImageJ software [33]. Calibration was performed with pictures of an object micrometer (0.01 mm) taken at the same photomicroscope and objective. The length of micro-aggregates was measured on their longest axis. In total, 10 micro-aggregates from each sample were measured (five micro-aggregates from each droplet) in three biological replicates, a total of 30 measurements from each fraction. The planktonic overnight cultures were measured in the same way.

### 2.5 Isothermal microcalorimetry

The metabolic rate of the different micro-aggregates fractions and the planktonic overnight cultures was evaluated by measuring heat flow using isothermal microcalorimetry. One day after filtration each micro-aggregate fraction and planktonic overnight culture were diluted to 10^6^ CFU/ml with sterile isotonic saline.

The calPlate™ (Symcell AB, Stockholm, Sweden) containing a total of 48 vials was prepared as previously described [34]. Just before loading the samples, 0.5 ml of each sample was added to 4.5 ml of TSB, resulting in a final concentration of 10^5^ CFU/ml of each sample in fresh media. Each vial was loaded with a 200 μl sample. Reference wells were loaded with sterile TSB [34]. The different size fractions in fresh TSB were added to the remaining vials with three to six technical replicates per sample. The calPlate™ was loaded to the calScreener™ with stepwise insertion to allow temperature equilibration to 37 °C [34] before the experiment was started and ran for approximately 14 h.

The isothermal microcalorimetry experiment was run with different batches of planktonic bacteria and micro-aggregates, to obtain three biological replicates from each fraction (Supplementary S1–S5).

### 2.6 Animal experiment

In total, six female Göttingen Minipigs were used (Ellegaard Göttingen Minipigs A/S, Dalmose, Denmark). The minipigs were 23–25 months of age and had an average weight of 44 kg, ranging from 41 to 49 kg. All minipigs had previously been used for breeding and had one to two litters. The minipigs arrived at the animal facility two weeks prior to the start of the study, allowing two weeks of acclimatization. All animals were housed in the same barrier stable but in separate pens, with a 12-h light/dark cycle and fed twice a day with a commercial pig diet *(Brogaarden Altromin, 9069 – Extrudate)* and had free access to tap water. The animals were handled, trained, and clinically evaluated daily during the experimental period. The experiment protocol was approved by the Danish Animal Experiments Inspectorate (license no. 2017-15-0201-01356).

The minipigs were allocated into three groups (n = 2, in each), receiving 10 μl of different inocula prepared as described above. Group A: overnight planktonic inoculum cultured in LB; 10^4^ CFU *S. aureus* (S54F9), acting as an infective control (this inoculum has been used in several studies of IAO) [[26], [27], [28], [29],^35^,^36^]. Group B: micro-aggregate-inoculum of the fraction obtained with filter size 5–15 μm cultured in TSB for seven days; 10^4 (5–15μm; 7 days old)^ CFU *S. aureus* (S54F9), Group C: sterile isotonic saline.

Inoculation, anesthesia and surgery were carried out as previously described by Jensen et al., 2017 [26]. In brief, anaesthetized animals were placed in the right lateral recumbency, exposing the medial side of the right tibia. An incision through the skin, subcutis and periosteum was placed approximately 10 mm distal to the proximal tibial growth plate. A 15 mm deep implant cavity (IC) was drilled into the tibia using a K-wire (4 mm in diameter). Inoculum were placed in the pre-drilled IC, and a sterile stainless-steel implant was inserted (diameter 2 mm, length 10 mm). Following implant insertion, the periosteum, subcutis and cutis were sutured in layers. All minipigs received daily treatment with non-steroidal anti-inflammatory drugs (NSAIDs) after surgery and throughout the experimental period. Seven days after surgery, the minipigs were euthanized by an intravenous overdose of pentobarbital 400 mg/ml. Postmortem computed tomography, macroscopic pathology, microbiology, and histopathology were performed.

### 2.7 Computed tomography (CT) and macroscopic pathology

CT-scanning of the right tibia was performed following euthanasia and implant removal using a single slide computed tomography (CT) scanner (Siemens Somatom Emotion; Siemens, Erlangen, Germany). The scans were performed in a cranio-caudal direction and with a slide thickness of 2 mm (kV = 130 and mAs = 55). A standard soft tissue algorithm (B80s) was used for reconstructions. A blinded evaluation was performed by a single assessor using the software system Osirix Lite (OsiriX, Bernex, Switzerland) [26]. Assessment of the presence of sequesters (yes/no), the degree of sclerosis surrounding the IC (mild/moderate/massive), the presence of osteolysis (yes/no) and measurements of the volume (cm^3^) of the IC was recorded.

The animals were necropsied, as previously described [26]. The surgical wound was inspected, and cutis and subcutis were opened in separate layers. Samples from subcutis were collected for microbiology using sterile surgical equipment. The periosteum was opened, and the position of the implant was identified. The right hind leg was cut off in the stifle joint, and the right tibia was sectioned sagittally through the IC, to allow evaluation of bone lesions surrounding the IC. The following findings were registered: purulent subcutaneous inflammation (yes/no), purulent exudate in the bone surrounding the IC (yes/no) and necrosis of the bone (yes/no). A combined lesions score was calculated for the registered CT and macroscopic pathology findings by yes = 1 and no = 0, resulting in a maximum score of 5.

The left tibial bone was sectioned correspondingly to the right as control. The abdomen and thorax were cut open and all organs were evaluated *in situ*. Samples from the liver, lung and right kidney were collected for histology.

### 2.8 Microbiology

Tissue samples from subcutis related to the wound, the peri-implanted pathological bone area (PIBA), and the right caudal lung lobe were collected sterilely, as well as a swab from the IC. Swabs and soft tissue samples were inoculated on blood agar plates and incubated at 37 °C for 24 h under normoxic conditions. Morphological distinct colonies were selected and identified by Matrix-assisted laser desorption ionization time-of-flight mass spectrometry (MALDI-TOF MS) (Vitek MS RUO, bioMérieux, Marcy-l'Etoile, France) [36]. Bone samples were aseptically homogenized and serial diluted in sterile saline before 100 μl were plated on blood agar plates, with one replicate per sample. The plates were incubated at 37 °C for 24 h under normoxic conditions. Morphologically distinct colonies were counted to estimate CFU/mL and re-streaked to obtain pure cultures for identification with MALDI-TOF MS. During necropsy, the implants were collected from the ICs using sterile surgical equipment, placed in cryotubes and covered with 1 ml sterile isotonic saline. All implants were sonicated in an ultrasound bath to detect any bacterial attachment and biofilm formation on the implants [35]. The sonication protocol has recently been published [37]. All the microbiological evaluations were performed blinded.

### 2.9 Histopathology and immunohistochemistry (IHC)

All tissues were placed in 10 % buffered formalin for seven days. Following fixation the osseous tissue was decalcified in a solution containing 3.3 % formaldehyde and 17 % formic acid for six weeks. After fixation or decalcification, all samples were trimmed to representative sections and processed through graded concentrations of alcohol and xylene, embedded in paraffin and sectioned. All sections of 4–5 μm thickness were stained with Hematoxylin & Eosin (HE). Additionally, bone sections from the right tibia were stained with IHC using antibodies towards *S. aureus* [29] and MAC387 (calprotectin) located in macrophages, monocytes and granulocytes [38].

The IC was identified, and the surrounding PIBA was evaluated for the presence of inflammatory cells, edema, fibroplasia or fibrosis, new-formed trabecular bone, active osteoclasts and bacteria. The neutrophils were counted using the method developed by Morgenstern et al., 2018 [39]. Ten high power fields (HPF) with high concentrations of neutrophils were identified, and within these, all clearly identifiable neutrophils were counted at 400x magnification, with a maximum of 10 neutrophils per HPF. The average number of neutrophils per HPF resulted in one of the following scores: 0 = no neutrophils, 1 = ≤one neutrophil per HPF, 2 = one to five neutrophils per HPF, 3 = >five neutrophils per HPF.

All HE-stained slides of the right tibial bones were scanned to digital slides (Zeiss Axioscan.Z1 microscope slide scanner, Zeiss, Oberkochen, Germany) with a 20x/0.8 objective. The scanned slides were evaluated using the Qupath software version 0.4.2 [40]. The PIBA area was outlined and measured (mm^2^) using the “wand” –and “free-hand” brush annotation tools. All newly formed trabecular bone present within PIBA were identified and measured in the same way.

Histological sections from the lung, liver, and kidney were evaluated for signs of pathological changes.

### 2.10 Statistics

All statistical calculations were performed in GraphPad Prism version 10.0.1 for Windows (GraphPad Software Inc., San Diego, CA, USA). P-values of ≤0.05 were considered statistically significant. The size measurements of the micro-aggregates were analyzed for normality and lognormality, followed by a one-way ANOVA analysis and Dunn's multiple comparisons test. The thermograms (metabolic rate measurements) (Supplementary S1–S5) for each vial were analyzed, and the time-to-peak metabolic activity of the thermograms found, resulting in three to six time-to-peak measurements for each fraction. All time-to-peak results were analyzed by One-way ANOVA followed by Tukey's multiple comparisons test.

## 3 Results

### 3.1 Filtration by cell strainers results in size separation of *S. aureus* micro-aggregates

**Fig. 3:**
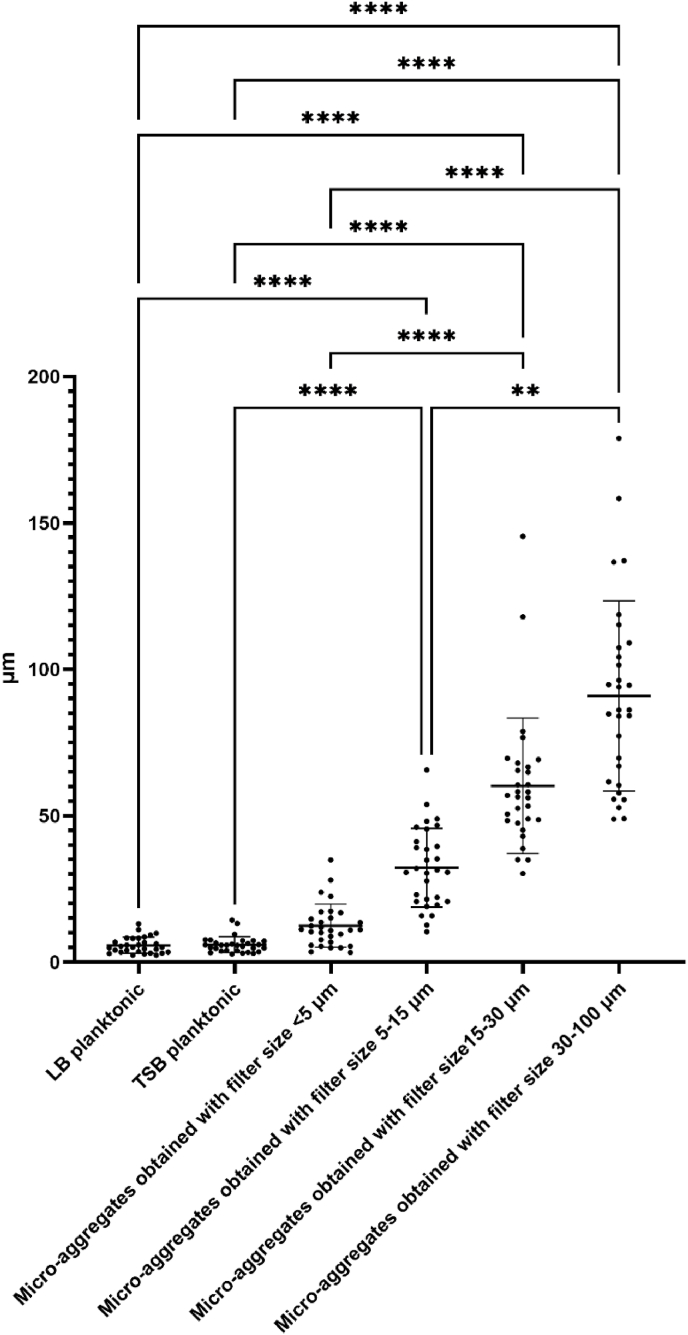
Measurements of the length of aggregated bacteria in each fraction, with means and SD for each fraction, including the two planktonic overnight cultures. **p ≤ 0.01, ****p ≤ 0.0001.

**Fig. 4:**
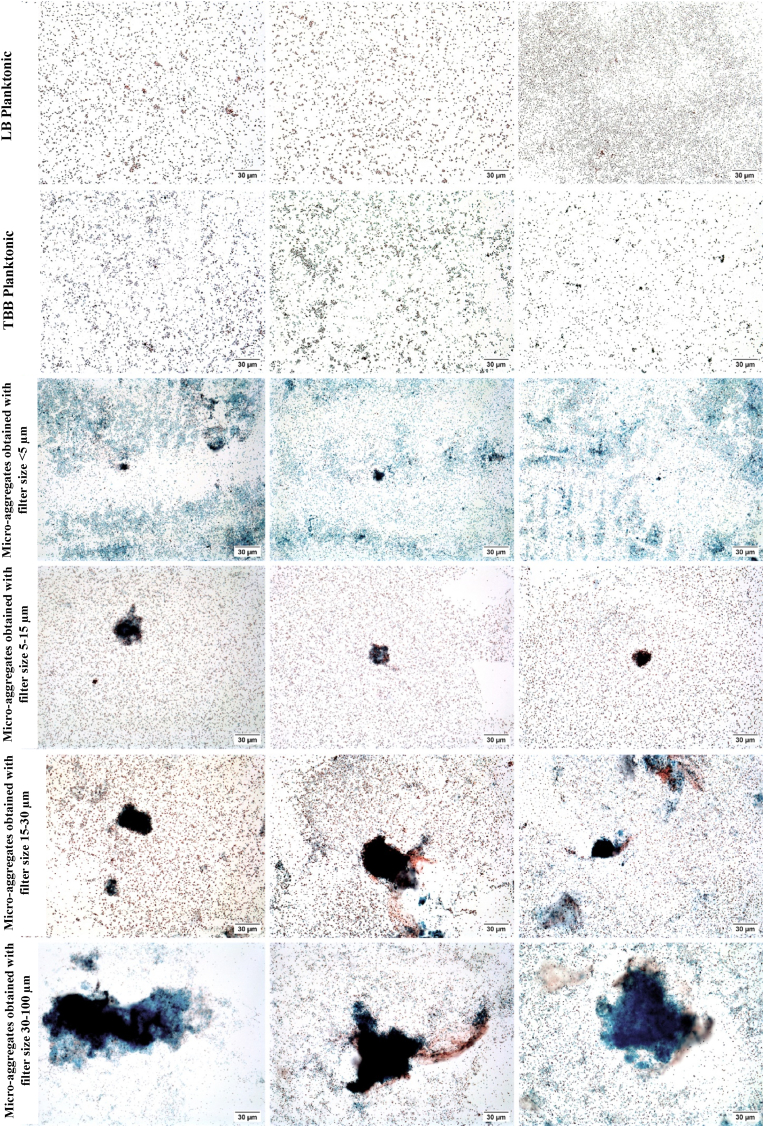
Pictures of the seven-day-old micro-aggregates from each fraction compared to planktonic bacteria cultured in Lysogeny Broth (LB) and Tryptone Soya Broth (TSB) for 24 h. The micro-aggregates are presented as dark red-brown clusters of immunohistochemistry (IHC)-positive *S. aureus* surrounded by Alcian blue-stained saccharides of extracellular matrix. Many single scattered planktonic bacteria surround the micro-aggregates. (For interpretation of the references to color in this figure legend, the reader is referred to the Web version of this article.)

Light microscopy confirmed that *S. aureus* strain S54F9 produced biofilm micro-aggregates when grown in TSB for seven days. Measurements of the micro-aggregates on their longest axis revealed that the developed filtration protocol does result in separation of micro-aggregates into significantly different sizes (Fig. 3). However, the mean length of the three smallest micro-aggregate fractions exceeds the pore size of the filters used, resulting in aggregates larger than defined by the filter sizes i.e. micro-aggregates obtained with the filter size <5 μm having an actual mean length of 12,5 μm, micro-aggregates obtained with the filter size 5–15 μm having an actual mean length of 32 μm, micro-aggregates obtained with the filter size 15–30 μm having an actual mean length of 60 μm, and micro-aggregates obtained with the filter size 30–100 μm having an actual mean length of 90 μm (Fig. 3). The micro-aggregates were seen as clusters of *S. aureus* IHC positive, red-brown-stained cells surrounded by blue-stained polysaccharides of the extracellular matrix (Fig. 4). Some bacteria also stained blue due to the polysaccharides on their cell surface. Alongside micro-aggregate formation, all fractions had a high amount of single planktonic bacteria (Fig. 4). In the smallest fraction (obtained with filter size <5 μm), only a few micro-aggregates were observed while most of the bacteria were organized as single cells, together with a high amount of blue stained polysaccharides. The planktonic overnight cultures in LB and TSB showed a higher density of bacteria than seen in the micro-aggregated fractions, with the bacteria mainly situated as single bacterial cells with a few bacterial cells clustered together. However, these small clusters showed reduced, blue-stained extracellular matrix or polysaccharides.

### 3.2 Micro-aggregated bacteria showed a delayed time-to-peak metabolic activity

Isothermal microcalorimetry, measuring heat flow over time, showed a modest delayed mean time-to-peak metabolic activity of all the micro-aggregate fractions compared to the planktonic overnight cultures (Table 1). A significant difference (Table 1) was seen between the mean time-to-peak of the planktonic LB culture compared to all micro-aggregate fractions, while no significant difference was found between the planktonic LB culture and the planktonic TSB culture. Furthermore, a significant difference was also seen in mean time-to-peak between the micro-aggregates obtained with filter size <5 μm and the planktonic TSB culture (p < 0.01) and the two micro-aggregate fractions obtained with filter size 15–30 μm (p < 0.05) and filter size 30–100 μm (p < 0.05), respectively. In summary, when comparing the two *in vivo* inoculums, i.e., the LB planktonic overnight culture and the seven days old micro-aggregate fraction obtained with filter size 5–15 μm, the micro-aggregate inoculum showed significantly delayed time-to-peak metabolic activity of 0.7 h compared to the planktonic inoculum.

**Table 1:** Time-to-peak heat production measured by isothermal microcalorimetry.

	Number of measurements	Mean Time-to peak (hour)	[Min-Max] (hour)	SD (hour)	SEM (hour)	p-value in comparison to LB planktonic bacteria	95 % CI of difference
LB planktonic	19	6.3	5.2–7.3	0.84	0.19	–	–
TSB planktonic	18	6.6	5.6–7.2	0.61	0.14	NS	−0,97 to 0,19
Micro-aggregates obtained with filter size <5 μm	26	7.4	6.3–8.8	0.94	0.19	p < 0.0001	−1,7 to −0,60
Micro-aggregates obtained with filter size 5–15 μm	27	7.0	6.6–7.3	0.24	0.045	p < 0.001	−1,28 to −0,22
Micro-aggregates obtained with filter size 15–30 μm	28	6.8	6.4–7.7	0.41	0.078	p < 0.05	−1,09 to −0,04
Micro-aggregates obtained with filter size 30–100 μm	25	6.8	6.1–7.3	0.39	0.078	p < 0.05	−1,09 to −0,01

SD = Standard deviation, SEM = Standard error of mean, CI = Confidence interval, LB = Lysogeny Broth, TSB = Tryptone Soya Broth.

### 3.3 Clinical observations

All animals were able to move freely and use the inoculated leg, but lameness in varying degrees was present in all the animals. One of the animals in Group B showed increasing lameness and impaired ability to stand three days after surgery and was treated with an intra-muscular injection of 0.1 mg/kg buprenorphine (0.3 mg/ml). After two times of treatment, the animal recovered.

### 3.4 Gross pathology and computed tomography findings

Positive signs of osteomyelitis, i.e., osteolysis, sequesters, bone necrosis and pus in the bone were seen in Group A animals, while only one animal from Group B presented with osteolysis as the only positive sign of osteomyelitis (Table 2). However, both Group B animals showed macroscopic signs of infection in the soft tissue in the form of subcutaneous abscesses. The animals in Group C showed no positive signs of infection in soft tissue or bone, and the degree of sclerosis was more pronounced in the Group C animals than in the Group A and B animals, indicating a stronger healing response in the mock-infected group. The 3D-estimated IC volumes and bone lesions were increased in Groups A and B compared to Group C (Table 2).

**Table 2:** Gross pathology evaluated by Computed Tomography scanning and macroscopic lesion assessment at necropsy.

		Computed Tomography scanning results				Macroscopic pathology assessment			**Total Pathology Score
Animal	Group	*Volume (cm^3^)	Sclerosis	Osteolysis	Sequesters	Subcutaneous pus	Bone Necrosis	Pus in the bone	
1	A: *S. aureus* planktonic	0.7219	Mild	Yes	Yes	Yes	Yes	Yes	5
2	A: *S. aureus* planktonic	0.2470	Moderate	Yes	Yes	Yes	No	Yes	4
5	B: *S. aureus* micro-aggregates	0.2536	Mild	Yes	No	Yes	No	No	2
6	B: *S. aureus* micro-aggregates	0.2526	Mild	No	No	Yes	No	No	1
3	C: Saline	0.1446	Massive	No	No	No	No	No	0
4	C: Saline	0.1325	Massive	No	No	No	No	No	0

*3D-estimated volumes of the implant cavities measured the software system Osirix Lite (OsiriX, Bernex, Switzerland) [26]

** Total Pathology score, calculated by No = 0 and Yes = 1 for each score, from both Computed Tomography scanning results and Macroscopic pathology assessment, without sclerosis and Volume, with a total maximum score of 5.

No lesions were observed in any of the animals' thoracic and abdominal organs.

### 3.5 Infection outcomes and microbiology

All lung and blood samples were sterile, i.e., showing no signs of systemic spread of the bacteria in either of the animals. *S. aureus* was isolated from soft tissues, bones, and implants in all Group A and B animals (Table 3). *Staphylococcus chromogenes,* a common skin inhabitant in pigs [41], was isolated from soft tissue and implants in both Group C animals, indicating intra-operative contamination.

**Table 3:** Bacteriology from soft tissue, bone, and implants.

Animal	Group	Subcutis tissue	Implant cavity swab	Bone – Quantified (CFU/g)	Implants – Quantified (mean CFU/ml)
1	A: *S. aureus* planktonic	*S. aureus*	*S. aureus*	*S. aureus* – 8.0 × 10^8^	*S. aureus* – 7.0 × 10^5^
2	A: *S. aureus* planktonic	*S. aureus*	*S. aureus*	*S. aureus* – 7.6 × 10^4^	*S. aureus* – 2.9 × 10^6^
5	B: *S. aureus* micro-aggregates	*S. aureus*	*S. aureus*	*S. aureus* – 3.4 × 10^6^	*S. aureus* – 7.5 × 10^3^
6	B: *S. aureus* micro-aggregates	*S. aureus*	*S. aureus*	*S. aureus* – 4.6 × 10^5^	*S. aureus* – 2.4 × 10^6^
3	C: Saline	*S. chromogenes*	*S. chromogenes*	Sterile^a^	*S. chromogenes* – 1.5 × 10^2^
4	C: Saline	*S. chromogenes*	*S. chromogenes*	Sterile^b^	*S. chromogenes* – 1.6 × 10^4^

a Below detection limit <1 CFU/0.02 g

b Below detection limit <1 CFU/0.0005 g

### 3.6 Histopathology and immunohistochemistry (IHC) findings

Histopathologically, all bone lesions showed necrotic bone tissue and cellular debris within IC adjacent to PIBA. In Group A animals, the IC was irregular, and PIBA consisted of a layer of fibroblasts intermingled with necrotic bone, neutrophils, and mononuclear cells, sometimes in accumulations. A narrow brim of newly formed trabecular bone with active osteoblasts and multiple active osteoclasts was seen outside this layer. Red intra –and extracellular *S. aureus-*positive bacteria were seen inside PIBA in both animals of Group A.

The bone lesions of Group B animals revealed a more regular IC outline. The outermost layer of PIBA consisted mostly of proliferating fibroblasts and necrotic bone as seen in Group A, but only one of the Group B animals presented with infiltration of neutrophils, mononuclear cells, and bacteria within PIBA. Bone remodeling with new osteoid formation, active osteoblasts and multiple osteoclasts were seen to a greater extent than observed in Group A animals.

No Group C animals showed signs of inflammatory cell infiltrations or bacteria within PIBA. For one Group C animal, only the bottom of PIBA and not the IC was included on the histological section. Therefore, measurements and quantification of IC, PIBA and newly formed osteoid, respectively, were not possible for this animal.

**Fig. 5:**
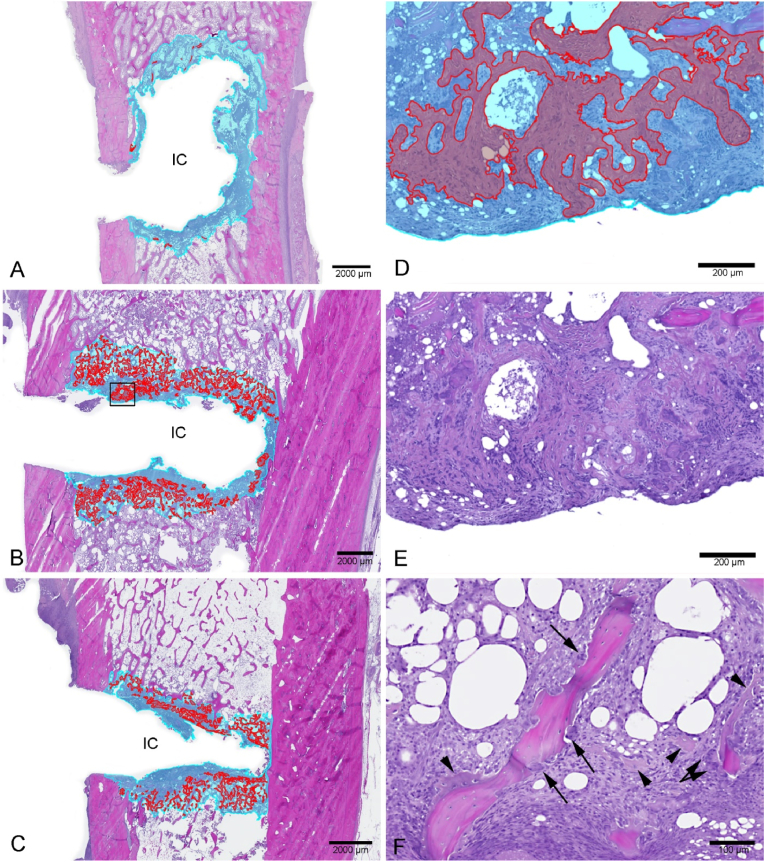
Presentation of the histological bone sections, with the peri-implant pathological bone area (PIBA) marked in turquoise, surrounding the implant cavity (IC). Inside PIBA new bone, i.e. osteoid is marked in red. A: Pig inoculated with planktonic bacteria. B: Pig inoculated with micro-aggregates of the 5–15 μm fraction. C: Pig inoculated with sterile 0.9 % saline. D: Close up from the box in B, of the new bone. E: Same picture as D but without the annotations. F: Active osteoclasts (arrows) and the newly formed osteoid (arrowheads). (For interpretation of the references to color in this figure legend, the reader is referred to the Web version of this article.)

No differences in IC or PIBA size were seen between the groups. However, an increased healing response was seen in the animals in Group B and C, as they had 10-70 times more newly formed trabecular bone within PIBA, and thereby, the ossification front was situated closer to the IC interface than in Group A animals (Fig. 5 and Table 4).

**Table 4:** Summary of the results of the histological examination of the right tibial bones.

Animal	Group	NG score	Number of IHC *S. aureus* positive colonies	IC area (mm^2^)	PIBA area (mm^2^)	New formed bone in PIBA (mm^2^)	% New bone of PIBA	Mean* distance from IC to new bone (μm)
1	A: *S. aureus* planktonic	3	>100	43.27	26.27	0.09	0.3	445.5
2	A: *S. aureus* planktonic	3	>100	13.82	20.07	0.10	0.3	697.5
5	B: *S. aureus* micro-aggregates	3	>100	30.85	29.25	1.26	4.3	45.5
6	B: *S. aureus* micro-aggregates	1	0	31.74	28.86	6.10	21.2	19.5
3	C: Saline	1	0	21.68	22.06	3.36	15.3	10.0
4	C: Saline	1	0	NE	NE	NE	NE	NE

NE = could not be evaluated on the slide, NG = neutrophil granulocyte, IHC = immunohistochemistry, IC = implant cavity, PIBA = peri-implanted pathological bone area. * Mean of the two shortest distances from the border of IC to the nearest newly formed bone.

Lesions were not present in the left tibial bone, the lymph nodes, lung, kidney, or liver in any of the animals.

## 4 Discussion

The present study shows that formation and size separation of low-metabolism bacterial micro-aggregates is possible using low-technical equipment for culturing and filtration [31]. Furthermore, low metabolic bacterial micro-aggregates can cause infection with microbiological re-isolation from both implants and surrounding tissue when used as inoculum in a porcine IAO model. The micro-aggregate inoculum caused a less aggressive IAO than the planktonic counterpart, primarily seen by pronounced osteoid formation (i.e., signs of healing). This suggests that low metabolic micro-aggregate-based inoculums are clinically relevant when investigating slowly developing, chronic IAI in animal models.

The filtration method successfully separated micro-aggregates into different size fractions, but inconsistencies were seen between filter pore size and the measured micro-aggregate end-size. Therefore, the micro-aggregate fraction obtained with filter size 5–15 μm, which was used as inoculum in the IAO pig model, had a mean length of 32 μm, just surpassing the mean size of human skin surface aggregates. The discrepancy in measurement and filter size might be due to two different factors: First, the filtration only occurs in two dimensions, making it possible for micro-aggregates to pass if their length and width can go through the pores and still be larger than the pore size in their height. Second, when preparing the micro-aggregates for visualization at the light microscope, a cover glass is placed upon the objective glass, which results in the squeezing of the micro-aggregates and, thus, elongation in two dimensions. More advanced microscopic techniques, such as confocal microscopy, might be considered for more precise micro-aggregate size measurements and visualization of their 3D structure. However, the combined staining technique and visualization by light microscope proved useful as proof of concept and quick evaluation.

The filtration process did not remove all the planktonic bacteria from the micro-aggregate fractions, and large quantities of planktonic bacteria were observed between the micro-aggregates. This might contribute to variance within the micro-aggregate fractions. A second filtration step could possibly diminish the proportion of planktonic bacteria in the larger fractions, and this should be applied as a refinement of the method in future studies.

However, the mixed bacterial population on the human skin may be better represented by such a mixed population of low metabolic micro-aggregates as well as single scattered bacteria [9].

The number of bacteria in the micro-aggregate fraction obtain with filter size 5–15 μm was evaluated by sonication. A previous study showed that aggregates could be separated through sonication of 75 % power [22]. However, in this present experiment, the sonication of up to 90 % power did not affect CFU count of the micro-aggregate fraction obtained with filter size 5–15 μm, suggesting either no separation of the micro-aggregates and that the effect of sonication might be strain-specific, and, therefore, not applicable to the strain used in this experiment, or an already dominance of single bacterial cells in the fraction. Determination of the number of viable bacteria in aggregates is challenging, and the CFU count method may be used to indicate the concentration of bacteria; however, it poses a risk of underestimation. However, a recent study showed a surprisingly good correlation between CFU counts and DNA content of viable bacteria in both aggregates and single-celled populations [31].

The heat flow measurements, representing the metabolic activity, showed a delayed time-to-peak of all the micro-aggregate fractions compared to the planktonic LB culture, with smaller micro-aggregates showing a slower time-to-peak. Since a significant difference between the TSB planktonic bacteria and the micro-aggregates fraction obtain with filter size <5 μm, in the time-to-peak heat-production was observed, but no significant difference in their size could be measured, it may be plausible to suggest that it is the age rather than the size that influences decreased metabolic activity of the bacteria. However, the micro-aggregates obtained with filter sizes 15–30 μm and 30–100 μm, with the same age as the fraction obtained with filter size <5 μm, had a similar time-to-peak metabolic activity as the planktonic TSB bacteria, which was an unexpected finding since larger aggregates were thought to resemble mature biofilm more i.e. being less metabolic active. Again, this unexpected finding raises the question of whether it is smaller size rather than the age, that influences decreased metabolic activity of the bacteria, and supports the choice of smaller aggregates as representative of skin flora and less metabolically active bacteria. The high sensitivity of isothermal microcalorimetry makes it important to work with the same number or concentrations of bacteria when comparing thermograms [34]. However, as mentioned above, the determination of the bacterial count of the micro-aggregates is a challenge and may be estimated falsely to low by the CFU count method. Considering this, previous studies have shown that high bacterial counts decrease time-to-peak measurements [42,43]. Despite potential inaccuracies in CFU count, which, if imprecise, is assumed to be falsely too low, the micro-aggregates still exhibited increased time-to-peak compared to planktonic overnight cultures, assumably being age rather than size-related. How old the bacterial cultures should be to gain this decreased metabolic activity could be interesting to investigate further.

Even though the decreased metabolic activity in the bacteria appears to be age-dependent rather than affected by size or degree of aggregation, the size of the aggregates is known to influence the cellular response of the immune system. Previously it was found that single neutrophils are limited to phagocytize aggregated bacteria of 5–10 μm in diameter or smaller, while several neutrophils are needed to phagocytose larger aggregates [14]. The size of the aggregates impairs the immunological clearance of the bacteria, and together with the decreased metabolic activity of the aggregates, this may result in a slower initiation of the infection and a reduced inflammatory response combined with their increased resilience towards antimicrobials [6].

Most animal models of IAO are inoculated with high concentrations of bacteria (10^2^–10^9^ CFU) [3,44,45] to ensure all inoculated animals develop infection and thereby increase the reproducibility of the model. However, in humans, only about 1–16 % of the patients receiving an orthopedic implant develop an infection [1,46], and the concentration of bacteria causing the infections is believed to be much lower [47]. Since it is important with reproducible animal models where all inoculated animals develop infections, both to trust the findings when testing new treatment strategies and to reduce the number of experimental animals, the concentration of bacteria used for inoculation might still need to be artificially high. However, other considerations, such as the metabolic state [5] and the organization of the bacteria [6], might be taken into account to improve the realism of the inoculum.

### 4.1 Additional finding

Maybe a new denoting method should be applied to inoculums since CFU alone does not describe the size and age, which, according to the present study, are important factors that can be varied widely. This paper proposes a new definition where both concentration by CFU count as well as size and age in the form of culturing time is included in the following way (Fig. 1):

Concentration ^(size; age)^∼10^4 (5–15 μm; 7 day)^ CFU

## 5 Conclusion

The main finding of the present study was that the pathology due to low metabolic micro-aggregates differed from the response observed with planktonic bacteria. The planktonic inoculum caused an acute bone infection with a high number of neutrophils, mononuclear cells, and bacteria, together with fibroplasia and osteolysis. In contrast, the micro-aggregated bacteria caused an altered bone response, more similar to what is seen in chronic cases of osteomyelitis with both inflammatory cells and bacteria but also with bone modelling, including pronounced osteoid formation. Clinically, chronic osteomyelitis and other types of bone infections shows bone modelling and healing, although clearly not efficient or complete, it results in a slow growth of the infection. This could result in a more contained infection without pronounced systemic impact, and, therefore, the observed contrast within pathology could help explain why IAI often are slow-onset low-grade infections. The micro-aggregates seem to offer a new and clinically relevant form of bacterial inoculum for animal models of IAI. However, the current *in vivo* study was limited to a small sample size, and to investigate and support these findings, further larger-scale *in vivo* studies will need to be conducted.

The present paper introduces the concept of micro-aggregates and their use as inoculum in animal models of IAI. Micro-aggregates represent a new and interesting inoculum form which seems to cause an altered tissue response in comparison to planktonic counterparts. However, further work with refining the size separation according to applied filter sizes, as well as eliminating the amount of planktonic bacteria would strengthen the definition of the fractions as being pure micro-aggregates. Such an optimization would also make the interpretation of the *in vivo* effects more clear. Additional investigation of metabolic activity and how it relates to age vs. size of the aggregates, as well as the effect of the different culture medias will also add in to improve understanding of the nature of micro-aggregates. These points will be of the highest relevance if the micro-aggregates are to be established as an equal alternative inoculum to the planktonic and surface attached biofilm counterpart.

## Funding

This study was financed by grant no. R345-2020-1674 from the 10.13039/501100003554Lundbeck Foundation and grant no. NNF19OC0056411 from the Novo Nordisk Foundation, Challenge Program.

## CRediT authorship contribution statement

**Katrine Top Hartmann:** Writing – review & editing, Writing – original draft, Visualization, Resources, Project administration, Methodology, Investigation, Formal analysis, Conceptualization. **Regitze Lund Nielsen:** Writing – review & editing, Methodology, Investigation. **Freja Cecilie Mikkelsen:** Writing – review & editing, Resources, Methodology, Investigation, Formal analysis. **Bent Aalbæk:** Writing – review & editing, Resources, Methodology, Investigation. **Mads Lichtenberg:** Writing – review & editing, Resources, Methodology, Investigation, Funding acquisition, Formal analysis, Conceptualization. **Tim Holm Jakobsen:** Writing – review & editing, Methodology, Funding acquisition, Conceptualization. **Thomas Bjarnsholt:** Writing – review & editing, Methodology, Funding acquisition, Conceptualization. **Lasse Kvich:** Writing – review & editing, Resources, Methodology, Investigation, Formal analysis. **Hanne Ingmer:** Writing – review & editing, Resources, Methodology. **Anders Odgaard:** Writing – review & editing, Conceptualization. **Henrik Elvang Jensen:** Writing – review & editing, Supervision, Methodology, Conceptualization. **Louise Kruse Jensen:** Writing – review & editing, Resources, Project administration, Methodology, Investigation, Funding acquisition, Formal analysis, Conceptualization.

## Declaration of competing interest

The authors declare the following financial interests/personal relationships which may be considered as potential competing interests: Louise Kruse Jensen reports financial support was provided by 10.13039/501100003554Lundbeck Foundation. Thomas Bjarnsholt reports financial support was provided by 10.13039/501100009708Novo Nordisk Foundation. If there are other authors, they declare that they have no known competing financial interests or personal relationships that could have appeared to influence the work reported in this paper.

## Data Availability

Data will be made available on request.
