# Supplementary material for: Bacterial micro-aggregates as inoculum in animal models of implant-associated infections
Source: Biofilm. 2024 May 9;7:100200. doi: 10.1016/j.bioflm.2024.100200 (PMC11128829; doi:10.1016/j.bioflm.2024.100200)
Supplement: Multimedia component 1 [file mmc1.docx]

# Supplementary

## Thermograms:

**Supplementary S1:** Thermogram from isothermal microcalorimetry experiment Run 1. The isothermal microcalorimetry experiment was run over five separate times with different batches of planktonic bacteria and micro-aggregates, to obtain three biological replicates from each fraction. Each fraction was measured in three to eight vials per run and a mean of these are presented at the graph.

**Supplementary S2:** Thermogram from isothermal microcalorimetry experiment Run 2. The isothermal microcalorimetry experiment was run over five separate times with different batches of planktonic bacteria and micro-aggregates, to obtain three biological replicates from each fraction. Each fraction was measured in three to eight vials per run and a mean of these are presented at the graph.

**Supplementary S3:** Thermogram from isothermal microcalorimetry experiment Run 3. The isothermal microcalorimetry experiment was run over five separate times with different batches of planktonic bacteria and micro-aggregates, to obtain three biological replicates from each fraction. Each fraction was measured in three to eight vials per run and a mean of these are presented at the graph.

**Supplementary S4:** Thermogram from isothermal microcalorimetry experiment Run 4. The isothermal microcalorimetry experiment was run over five separate times with different batches of planktonic bacteria and micro-aggregates, to obtain three biological replicates from each fraction. Each fraction was measured in three to eight vials per run and a mean of these are presented at the graph.

**Supplementary S5:** Thermogram from isothermal microcalorimetry experiment Run 5. The isothermal microcalorimetry experiment was run over five separate times with different batches of planktonic bacteria and micro-aggregates, to obtain three biological replicates from each fraction. Each fraction was measured in three to eight vials per run and a mean of these are presented at the graph.
